# Effect of Resistance Training on Hematological Blood Markers in Older Men and Women: A Pilot Study

**DOI:** 10.1155/2009/156820

**Published:** 2009-10-27

**Authors:** Florian Bobeuf, Mélissa Labonté, Abdelouahed Khalil, Isabelle J. Dionne

**Affiliations:** ^1^Research Centre on Aging, University of Sherbrooke, Sherbrooke, QC, Canada J1H 4C4; ^2^Faculty of Physical Education and Sport, University of Sherbrooke, Sherbrooke, QC, Canada J1K 2R1; ^3^Department of Medicine, Service of Geriatrics, Faculty of Medicine, University of Sherbrooke, Sherbrooke, QC, Canada J1K 2R1

## Abstract

The aim of this study was to examine the effects of resistance training on hematological blood markers in older individuals. Twenty-nine men and women participated to this study. Subjects were randomized in 2 groups: (1) control (*n* = 13) and (2) resistance training (*n* = 16). At baseline and after the intervention, subjects were submitted to a blood sample to determine their hematological profile (red blood cells, hemoglobin, hematocrit, platelets, leukocytes, neutrophils, lymphocytes, monocytes, mean corpuscular volume, mean corpuscular hemoglobin, mean corpuscular hemoglobin concentration, red cell distribution width). At baseline, no difference was observed between groups. Moreover, we found no significant difference after the intervention on any of these markers. A 6-month resistance program in healthy older individuals seems to have no beneficial nor deleterious effects on hematological blood parameters. However, resistance training was well tolerated and should be recommended for other health purposes. Further studies are needed to confirm these results in a large population.

## 1. Introduction

Aging is associated with a wide variety of changes, including modifications in blood profile: hematological and immunological functions are down-regulated, and susceptibility to develop various diseases (both infectious and noninfectious) is increased [1]. The fact that the aging process attenuates the ability to maintain an effective immune response is commonly accepted and is defined as immunosenescence. Cellular and biochemical mechanisms that enable to explain the immunosenescence are under investigations [2]. Changes in hematological parameters suggest that they could have a physiologic impact on immune defence [3].

Concomitantly, some interventions such as physical activity have been proposed to reverse or minimize these changes and to improve hematological parameters. Changes in blood hematological characteristics have been related to aerobic exercise, such as a decrease in hematocrit and an increase in hemoglobin content or platelet number in young and old individuals [4]. Moreover, moderate aerobic exercise was showed to produce a large impact on immune response [5].

On the other hand, acute resistance exercise was studied in young people, and an increase in leukocytes count with different variations between subpopulation of leukocytes (neutrophils, lymphocytes, and monocytes) was reported [6]. Furthermore, Ahmadizad and El-Sayed (2005) found transient changes in blood rheological variables after acute resistance exercise, and these changes could be attributed to exercise-induced alterations in hemoconcentration [7]. As such, plasma volume decreased leading to an increase in red blood cell count, haemoglobin, and hematocrit. In this sense, plasma viscosity increased immediately after acute resistance exercise and returned to normal values at the end of the recovery period [7]. However, a resistance training program (8 weeks) did not modify leukocyte counts (lymphocyte subsets) at rest in older sedentary adults [8]. In accordance, hematological parameters were unchanged by a resistance training program (12 weeks) in older men and women [9]. Nevertheless, the effect of long-term resistance training on hematological blood markers in older adults remains unknown. Thus the purpose of this randomized trial was to investigate whether 6 months of resistance training could improve hematological blood parameters in older men and women.

## 2. Methods

### 2.1. Subjects

Twenty-nine subjects (14 men and 15 women) aged 61–73 years (66.7 ± 3.7 years) were recruited with the use of advertisements in local newspapers to participate in a large scale study [10]. To be included in the study, subjects had to meet the following criteria: healthy, without major physical incapacity, sedentary, weight stable (±2 kg) for the last 6 months, nonsmoker, moderate drinker (max 15 g of alcohol/day, the equivalence of one alcoholic beverage/day), no medication that could influence glucose or lipid metabolism, absence of menstruation for the past, 12 months and no hormonal replacement therapy for women.

### 2.2. Study Procedures

A phone interview was conducted to screen for the aforementioned inclusion criteria. After explanations about nature and goals of the study, the subjects were invited for a visit at the Research Centre on Aging (Geriatric Institute of University of Sherbrooke). Upon arrival, they provided written informed consent, and a 12-hour fasting blood sample was obtained. At the end of visit, subjects were randomized in one of 2 groups: (1) control and (2) resistance training. All procedures and the protocol were approved by the Ethics Committee of the Geriatric Institute of University of Sherbrooke.

### 2.3. Resistance Training

Participants performed a resistance training program for 6 months. Each one-hour session comprised warm-up, exercises and recovery, including 8 exercises for the upper and lower body. Program was practiced three days (Monday, Wednesday, and Friday) per week and comprised 3 sets of 8 repetitions at 80% of 1-RM. To be included in final analyses, participants had to have participated in 85% of the sessions. All sessions were supervised by a kinesiologist.

### 2.4. Blood Collection and Biochemical Analyses

Blood samples were obtained in the morning after a 12-hour fasting. Plasma hematological profile (red blood cells, hemoglobin, hematocrit, platelets, leukocytes, neutrophils, lymphocytes, monocytes, mean corpuscular volume, mean corpuscular hemoglobin, mean corpuscular hemoglobin concentration, red cell distribution width) was analyzed in the clinical laboratory of the Geriatric Institute of University of Sherbrooke (Gen-S Hematology Analyzers, BeckmanCoulter, Mississauga, ON, Canada). Plasma albumin was analyzed by chemical technique [11] (Dimension Xpand plus, Siemens, Deerfield, IL, USA) in the Research Centre on Aging.

### 2.5. Statistical Methods

Descriptive statistics were used to present anthropometric data. Values are displayed as mean ± SD. Independent sample *t*-tests were used to compare groups at baseline. The effects of the intervention on all variables were assessed using repeated measures—analysis of variance (ANOVA) (time [0 and 6 month] × group [control and training]) when adjusting for gender. Independent sample *t*-tests were used to compare the percent change of blood cell variables between control and training groups. The percent change of the blood cell variables from baseline to 6-month follow-up was calculated as (Values_6month_ − Value_baseline_)/Value_baseline_ × 100. Paired *t*-tests were also used to examine difference between baseline and postintervention measurements in each group. Significance was set at *P* ≤ .05. All analyses were performed with the SPSS software version 15.0 (SPSS Inc., Chicago, IL).

## 3. Results

Age, height, weight, body mass index, fasting insulin, fasting glucose, and plasma albumin were not statistically different between control and resistance training groups at baseline (Table 1). Both control and resistance training groups exhibited no significant change in these variables after 6 months of intervention, even when gender was taken into account as a covariable.

 Red blood cells, hemoglobin, hematocrit, platelets, leukocytes, neutrophils, lymphocytes, monocytes, mean corpuscular volume, mean corpuscular hemoglobin, mean corpuscular hemoglobin concentration, and red cell distribution width were not statistically different between control and resistance training groups at baseline (Table 2). Both control and resistance training groups exhibited no significant change in these variables after 6 months of intervention, even when gender was taken into account as a covariable. Analyses on percent change of blood cell variables revealed no difference between groups. Finally, no difference between baseline and postintervention was found in each group.

## 4. Discussion

The aim of this pilot study was to examine the impact of intense resistance training on the hematological blood profile in older men and women. The major finding of this study is that this type of physical activity exerts no important impact on hematological parameters in older men and women.

 Physical training triggers a series of changes in the erythrocyte system of the peripheral blood cells that results in the elevation of the oxygen-carrying capacity of blood. The effects of endurance training on red blood cell indices are well documented and include an increased number of red blood cells [12]. Nevertheless, we did not find any significant changes in red blood cell count following resistance training in older adults. Because Ahmadizad and El-Sayed [7] and Hu et al. [13] demonstrated that resistance training can increase red blood cell counts in young adults, we believe that resistance training is an effective strategy to improve erythrocyte system. However, our results, together with those of Murray-Kolb et al. [9], suggest that aging may impair the capacity to respond to resistance training when considering red blood cell count.

 We also examined the count and proportion of leukocytes. Indeed, with acute resistance exercise, an increase in leukocytes number was observed in young adults [14] and in older women [15]. On the other hand, significant changes in leukocytes sub-populations were observed in young adults after an acute bout of resistance exercise [6]. However, our results suggest that a 6-month resistance training may have no beneficial impact on leukocyte neither number nor subpopulation in older men and women. Hence, there seems to be no change in older adults as to the number or proportions in sub-populations of leukocytes following resistance training. This could be due to the fact that resistance training does not solicit white blood cell or that older individuals are not sensitive to resistance training in terms of intensity, duration, frequency, or modality of exercise.

 The immune response (such as natural killer cell cytotoxic activity and lymphoproliferative response) is an important component in the susceptibility to develop various diseases [1]. Some studies using aerobic training show that it may alter the kinetics of immune response [3]. Moreover, it appears that long-term aerobic training interventions (more than 6 months) show the greatest effect, suggesting that a long period of time may be necessary before adaptations in immune function occur [3]. In this case, a 6-month period of resistance training could be insufficient to produce beneficial adaptations. However, we did not measure immune response or episodes and number of days of infection problems. We can only mention that our subjects did not complain about health problems of any kind during the intervention period. More studies are needed to examine the impact of resistance training on functional immunological parameters.

 Ahmadizad and El-Sayed [7] found transient changes in blood viscosity after an acute resistance exercise, concluding that this change could be attributed to exercise-induced changes in hemoconcentration. In fact, it was shown that plasma volume decreased leading to an increase in red blood cell concentration, haemoglobin, and hematocrit [7]. In this case, plasma viscosity increased immediately after resistance exercise and returned to normal at the end of the recovery period [7]. After 6 months of resistance training, our results showed no change in plasma albumin, hematocrit, red blood cell count, or plasma volume change, which are all implicated in blood viscosity. Thus, we believe that blood viscosity may not have been deteriorated. It is an interesting point since deleterious rheological changes are known to be a risk factor in cardiovascular complications and diseases [16]. In this case, being involved in a resistance training program should be recommended for healthy older adults, even if more studies are needed to examine this matter.

 Our study had several limitations that may explain the lack of effect on general hematological blood parameters. Although red blood cells, hemoglobin, platelets, and leukocytes parameters were within the lower limit of the normal, it is possible that because subjects were healthy and the sample size was small, it was not possible to produce significant effects. Nonetheless, even if subjects were healthy, they could have been at risk of developing some minor infections [1], and our results suggest that resistance training was not detrimental for immune functions in these older individuals. Finally, we did not measure lymphocyte subsets, which may have been useful in interpreting the results.

 To our knowledge, this is the first study to examine the effect of a 6-month resistance training program in older men and women. Our results demonstrate that intense resistance training seems to provide neither improvement nor detrimental effects on general hematological blood parameters in healthy older individuals. Nonetheless, further studies with a greater sample size are necessary to confirm these results.

## Figures and Tables

**Table 1:** Physical and biochemical characteristics of subjects by groups before and after intervention.

Variables (Mean ± SD)	Group 1 (*n* = 13; 7♀/6♂) control		Group 2 (*n* = 16; 8♀/8♂) resistance training	
	Baseline	Posttest	Baseline	Posttest
Age (years)	66.8 ± 4.0		66.6 ± 3.5	
Height (m)	1.62 ± 0.07		1.64 ± 0.07	
Weight (kg)	67.8 ± 8.7	67.9 ± 9.4	73.9 ± 11.2	74.3 ± 11.7
Body mass index (kg/m^2^)	25.6 ± 2.6	25.6 ± 2.6	27.2 ± 2.7	27.4 ± 3.0
Fasting insulin (pmol/L)	55.0 ± 24.1	59.7 ± 22.0	61.6 ± 25.8	64.3 ± 30.7
Fasting glucose (mmol/L)	4.9 ± 0.2	4.7 ± 0.4	4.8 ± 0.3	4.9 ± 0.8
Plasma albumin (mmol/L)	43.0 ± 1.3	43.6 ± 1.6	43.6 ± 1.9	43.2 ± 2.2

**Table 2:** Hematological parameters of subjects by groups before and after intervention.

Variables (Mean ± SD)	Group 1 (*n* = 13) control		Group 2 (*n* = 16) resistance training	
	Baseline	Posttest	Baseline	Posttest
Red blood cells (10^12^/L)	4.5 ± 0.3	4.4 ± 0.2	4.5 ± 0.5	4.4 ± 0.4
	♀: 4.4 ± 0.2	♀: 4.4 ± 0.1	♀: 4.3 ± 0.5	♀: 4.2 ± 0.4
	♂: 4.6 ± 0.4	♂: 4.5 ± 0.2	♂: 4.7 ± 0.4	♂: 4.6 ± 0.3
Hemoglobin (g/L)	136.6 ± 8.1	135.7 ± 6.8	138.6 ± 13.1	137.0 ± 13.9
	♀: 134.8 ± 6.5	♀: 133.7 ± 4.6	♀: 131.5 ± 10.8	♀: 128.3 ± 12.3
	♂: 138.6 ± 9.9	♂: 138.1 ± 8.6	♂: 145.8 ± 11.6	♂: 145.6 ± 9.6
Hematocrit	0.40 ± 0.02	0.40 ± 0.01	0.41 ± 0.03	0.40 ± 0.04
	♀: 0.39 ± 0.01	♀: 0.39 ± 0.01	♀: 0.39 ± 0.03	♀: 0.38 ± 0.03
	♂: 0.41 ± 0.02	♂: 0.40 ± 0.01	♂: 0.43 ± 0.03	♂: 0.42 ± 0.03
Platelets (10^9^/L)	246.8 ± 47.2	219.1 ± 53.0	226.1 ± 52.2	233.1 ± 61.8
Leukocytes (10^9^/L)	5.4 ± 1.2	5.4 ± 1.6	5.3 ± 1.2	5.2 ± 1.1
Neutrophils (%)	61.0 ± 6.8	62.3 ± 9.7	62.3 ± 9.7	63.1 ± 8.1
Lymphocytes (%)	32.6 ± 6.5	30.6 ± 8.0	31.3 ± 9.0	30.0 ± 7.7
Monocytes (%)	6.3 ± 2.4	6.2 ± 1.6	6.1 ± 2.3	6.3 ± 2.4
Mean corpuscular volume (F/L)	89.7 ± 2.3	89.6 ± 2.4	90.9 ± 3.0	91.1 ± 3.2
	♀: 90.1 ± 2.7	♀: 89.5 ± 1.8	♀: 90.3 ± 3.2	♀: 90.5 ± 3.7
	♂: 89.3 ± 1.8	♂: 89.6 ± 3.3	♂: 91.5 ± 3.0	♂: 91.8 ± 2.8
Mean corpuscular haemoglobin (pg)	30.1 ± 1.0	29.6 ± 3.3	30.6 ± 1.5	30.8 ± 1.1
Mean corpuscular hemoglobin concentration (g/L)	337.2 ± 6.0	339.3 ± 8.6	337.6 ± 7.6	338.1 ± 6.1
Red cell distribution width (%)	13.1 ± 0.6	13.2 ± 0.4	13.2 ± 0.5	13.4 ± 0.6
